# Maternal Feeding Practices among Children with Feeding Difficulties—Cross-sectional Study in a Brazilian Reference Center

**DOI:** 10.3389/fped.2017.00286

**Published:** 2018-01-04

**Authors:** Rachel H. V. Machado, Abykeyla M. Tosatti, Gabriela Malzyner, Priscilla Maximino, Cláudia C. Ramos, Ana Beatriz Bozzini, Letícia Ribeiro, Mauro Fisberg

**Affiliations:** ^1^Instituto PENSI, Hospital Infantil Sabará, Fundação José Luiz Egydio Setúbal, São Paulo, Brazil

**Keywords:** responsive caregiving, mothers, feeding practices, feeding difficulties, feeding complaints, children

## Abstract

**Background:**

Given the positive influence of responsive caregiving on dietary habits in childhood, to raise awareness of caregivers regarding their behavior is crucial in multidisciplinary care on infant feeding.

**Objectives:**

To identify the most common responsive and non-responsive feeding practices in mothers of children with feeding complaints, as well as to seek associations between practices and caregivers’ profile.

**Methods:**

Cross-sectional study with 77 children under 18 years old, with complaints of feeding difficulties. Data were collected during interviews with mothers: child age, gender, duration of exclusive breastfeeding, presence of organic disease, dynamics of bottle use, self-feeding practices and posture at meals, use of appropriate feeding equipment; basic information about the mothers (parity and level of education), caregiver feeding style, presence of coercive feeding, frequency and characteristics of family meals. Statistical analysis considered significance level at 5%.

**Results:**

The non-responsive profile predominated among mothers (76.2%, with the Authoritarian style being the most prevalent—39.7%). The responsive profile was characterized by absence of coercive feeding, stimulation of self-feeding practices, use of appropriate feeding equipment and meal environment, with interaction at meals. Non-responsive profile consisted of both inadequate environment and posture at meals, use of distraction and coercive feeding, lack of shared meals, and disregard for children’s hunger signals. Only the habit of sharing meals with children was associated with mothers’ profile, and considered a protection factor against non-responsive care (OR 0.23; 95% CI 0.06–0.88). Both Authoritarian (*p* = 0.000) and indulgent mothers (*p* = 0.007) breastfed exclusively for longer time than negligent ones. There was a higher level of interaction with children in “responsive” parental style (OR 0.056; *p* = 0.01) compared to other feeding styles.

**Conclusion:**

Results highlight the need for educational interventions focused on caregivers’ behaviors.

## Introduction

Caregivers’ feeding behaviors are an important influence in the formation of eating habits in childhood, given their role in the decisions about what, when, and where meals are offered; and in leaving children to decide whether to eat or not, as well as to the amount of intake in each meal (1). In this context, the term “responsive caregiving” consists of a set of behaviors that address caregivers’ attention and interest in the process of feeding their children, with respect for their hunger and satiety signals, and for their communication skills; in addition to stimulation toward effective and independent feeding processes (2).

Such influence in eating habits and nutritional status of children has been widely discussed over recent years, and its positive impact on consumption of healthy foods, availability, and intake of food groups and micronutrients, and improvement of healthy eating behaviors, social skills development, learning, and self-esteem have been described worldwide. The reduction in intake of sweets and sugar-sweetened beverages, obesity rates, sedentary activities, and psychosocial disorders in adolescence are also described as a consequence of responsive caregiving (3–7). The responsive caregiver tends to engage more in family mealtimes (8) and stimulates children’s autonomy and independence in all development spheres (9), whereas non-responsive caregivers tend to use authoritarian, indulgent, negligent, and/or coercive practices to feed children (such as restriction of food intake, pressure to eat, rewarding/blackmailing practices, offer of low-quality foods, punishments, and distractions during meals) (10); creating a relationship without reciprocity between both (2).

The relationship between caregivers and children with complaints of feeding difficulties (FD) is typically described by health professionals as non-responsive. FD are common and recurrent issues during early childhood, with prevalence range of 20–60%. With heterogeneous origins, they are characterized by behaviors such as multiple food aversions, total or partial food refusal, exacerbated food neophobia, limited intake of specific food groups, strong food preferences, delays in sucking, swallowing or chewing patterns, self-inflicted vomiting, tantrums, and other behaviors during meals (11). These complaints may be temporary or persistent, with potential implications in growth, development, and in the relationship with caregivers (12). Hence, it is natural to expect a non-responsive relationship between child and adult in such environments, which generates concern and anguish of family members and misbalance of family dynamics and relationship. Thus, to raise caregivers’ awareness constitutes an important step in the follow-up of these children.

In Brazil, studies that address responsive caregiving and FD are scarce need further investigation. Therefore, the purpose of this article is to identify the most common non-responsive and responsive maternal feeding practices in families with FD complaints, as well as to seek associations between practices and caregiving styles.

## Materials and Methods

### Study Design and Population

It is a cross-sectional study, carried out at the Centro de Dificuldades Alimentares (CDA), part of Instituto PENSI-Hospital Infantil Sabará-Fundação José Luiz Egydio Setúbal, located in São Paulo/Brazil. CDA is an outpatient service which follows children and adolescents between 0 and 18 years old with complaints of FD [except for the diagnosis of eating disorders according to DSM-5 (13)]. The population was assembled by convenience, with the inclusion of all patients followed in the service until data collection (*n* = 77, August, 2014 to January, 2016). All patients presented written consent forms signed by their responsible caregiver, after ethical approval of the project (CAAE 32939314.0.0000.5567; approval granted in 13/08/2014 under document n. 808.394).

### Data Collection

Data were collected from the interviews with patients’ mothers, as part of the service protocol, which consists of an appointment with a pediatrician, speech therapist, and nutritionist altogether, followed by a multidisciplinary discussion. FD are diagnosed as “Children with limited appetite,” “Agitated children,” “Phobic children,” “Misperception of caregivers,” “Picky eating,” and “Organic causes,” according to criteria suggested by Kerzner et al. (12). Families receives then a feedback, with indication of a therapeutic plan designed by each specialty (such as diet plans and nutritional education activities, medications, stimulation, and reestablishment of oral functions or even referral to other professionals from other areas). The guidelines used by each specialty were defined according to standards for age and are described by Maximino et al. (11). Data were collected at the initial appointment at CDA, and information was later extracted from records, of which, the following variables were selected and grouped according to the type of information:

- Children
  1. Personal data: age (in months, adjusted in case of preterm birth), gender, duration of exclusive breastfeeding (in months), and presence of organic disease associated with the feeding complaint. All children participating in the study had FD complaints, regardless of the type of complaint (partial or complete refusal to eat, picky eating, insufficient weight gain, low acceptance of food textures, etc.);
  2. Feeding skills: frequency of prolonged bottle use, self-feeding practices and posture at meals, and use of appropriate feeding equipment. The standards for feeding skills adopted at CDA follow current recommendations for proper development (14–18) and is described by Maximino et al. (11), as summarized below:
    - Utensils: bottle use up to 24 months (prolonged use after 24 months); full use of cutlery at 9 months of age;
    - Posture: use of booster seat or high chairs that promote a 90° inclination;
    - Self-feeding practices: to stimulate manipulation of foods as much as possible, giving children opportunity to self-feed with finger foods from 9 months onward;
- Maternal information: age (years), parity and education level, caregiving style according to Hughes et al. (19)—translated to Portuguese and validated by Fontanezi et al. (ahead of print), and use of specific coercive practices during meals (use of force and distractions). Caregiver styles are classified as follows below:
  1. Authoritative (responsive): high levels of affection and communication, as well as control and demands, serving the classical definition of responsive caregiving
  2. Three types of non-responsive profiles:
    - Indulgent: high levels of affection and communication, coupled with lack of control and demands. Caregivers are unlikely to establish rules or exercise control over the child’s behavior;
    - Negligent: low levels of demand and responsiveness. Caregivers with little involvement in tasks of raising and educating children;
    - Authoritarian: high levels of control and demand, and lack of affection and communication. Caregivers who tend to use coercive practices to moderate intolerable behaviors.
- Family routines: frequency and characteristics of shared family meals during the week (meal environment, presence of adults eating simultaneously, duration of meal in minutes, respect for signals of child hunger and satiety, and interaction between mother and child during the meal). The mother–child interaction was evaluated through the habit of talking with the child during meals [item 15 of the instrument used for parental classification (19)]. Meal environment was deemed adequate if provided opportunities for proper posture to eat and for children’s self-feeding practices (which require a table and chairs), as well as the opportunity to share meals with members of family and learn about feeding behaviors; hence compatible with kitchen and dining room environments (1).

### Statistical Analysis

After evaluating consistency of the data collected in Excel platform, statistical analysis was performed by SPSS v21 software. The descriptive analysis was performed by frequency of distribution (%) for categorical variables, and mean ± SD and quartiles for continuous variables. The four caregiving styles identified were later recoded into “responsive” and “nonresponsive” care (sum of the three remaining nonresponsive styles) to complement the analyses. For tests of association between variables and caregiving styles, the ANOVA test, Chi-Squared, and binary logistic regression were used. A significance level of less than 5% was considered.

## Results

**Figure 1:**
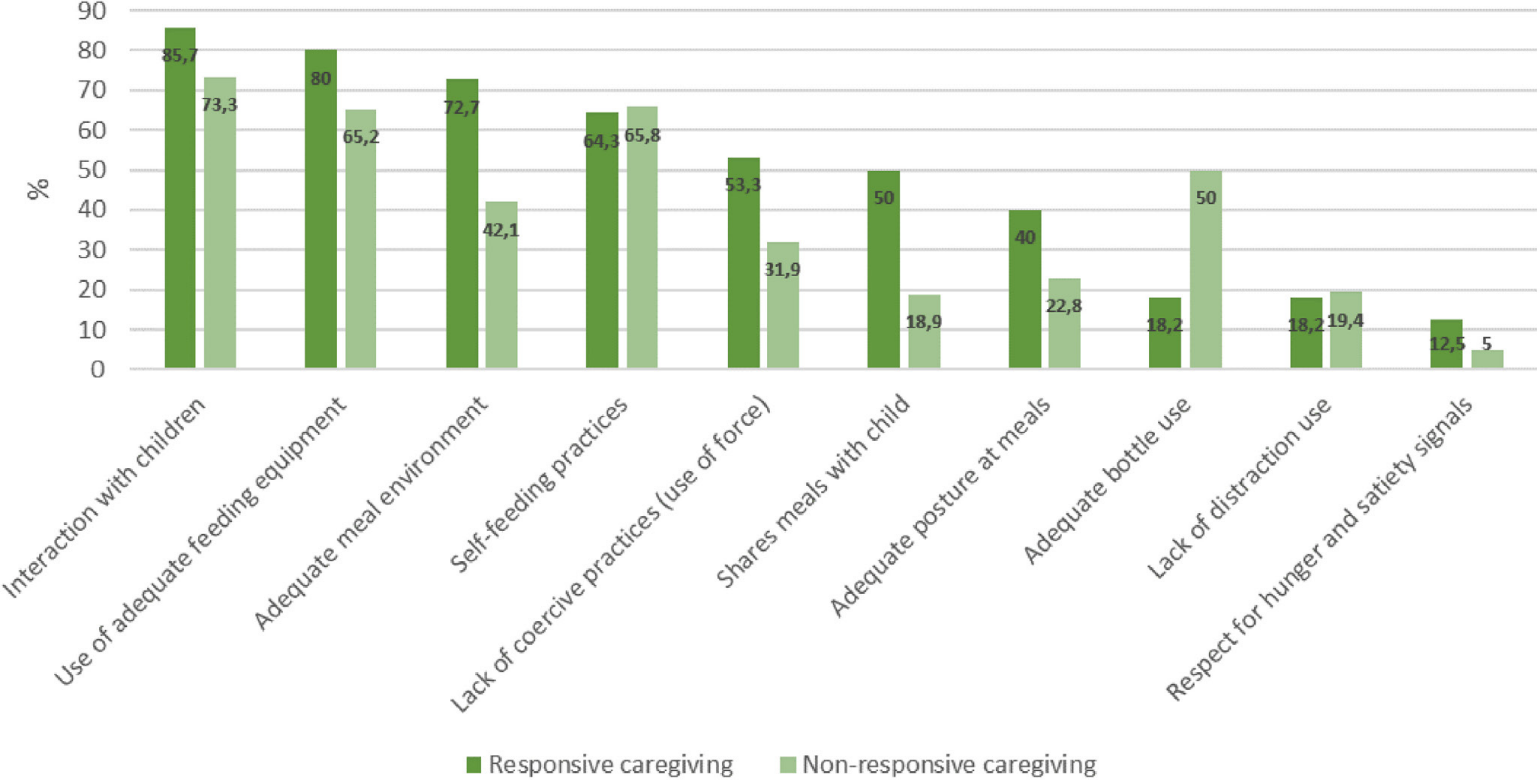
Frequency (%) of maternal adequate practices according to type of caregiving. Instituto PENSI, 2016.

General characteristics of the population are described in Table 1. Figure 1 describes the frequency of adequate practices reported by mothers, classified according to caregiving styles considered responsive and non-responsive. First, all mothers adopt adequate practices, although in different proportions according to their profile. Out of the ten practices evaluated, 60% are regularly adopted by half or more of the mothers considered as responsive caregivers; whereas in the non-responsive group, this fraction is reduced to 40%.

**Table 1:** General characteristics of the population.

Variable	Total population [% (*N*) or mean ± SD]
**Gender (*n* = 77)**	
Females	35.1% (27)
Male	64.9% (50)
Age (months) (*n* = 77)	44.1 ± 38.5 (p25% 19.50; p50% 33; p75% 55)
Duration of exclusive breastfeeding (months) (*n* = 70)	2.8 ± 2.7 (p25% 0; p50% 2;—p75% 6)
**Association with organic disease (*n* = 74)**	
Yes	32.4% (24)
No	67.6% (50)
**Bottle use (*n* = 52)**	
Inadequate (after 24 months of age)	57.7% (30)
Adequate	42.3% (22)
**Self-feeding practices (*n* = 62)**	
Yes	64.5% (40)
No	35.5% (22)
**Posture at meals (*n* = 74)**	
Adequate	27% (20)
Inadequate	73% (54)
**Use of proper utensils (*n* = 75)**	
Adequate	66.7% (50)
Inadequate	33.3% (25)
Maternal age (years) (*n* = 62)	36.1 ± 5.2 (p25% 33–p75% 40)
**Maternal level of education (*n* = 72)**	
High school	5.6% (4)
Superior	94.4% (68)
**Parity (*n* = 75)**	
Primiparous	78.7% (59)
Multiparous	21.3% (16)
**Coercive practices (use of force) (*n* = 73)**	
Yes	65.8% (48)
No	34.2% (25)
**Coercive practices (use of distractions) (*n* = 55)**	
Yes	81.8% (45)
No	18.2% (10)
Duration of meals (min) (*n* = 24)	47.9 ± 29.5 (p25% 30–p75% 57.5)
**Meal environment (*n* = 62)**	
Adequate	46.8% (29)
Inadequate	53.2% (33)
**Presence of adults at meals (*n* = 62)**	
Yes	27.4% (17)
No	72.6% (45)
Shared meals per week (*n* = 72)	2 ± 3.2 (p25% 0–p75% 5)
**Respect for signals of hunger and satiety (children older than 24 months) (*n* = 42)**	
Yes	2.4% (1)
No	97.6% (41)
**Interaction mother–child (*n* = 65)**	
Yes	76.9% (50)
No	26.1% (15)
**Caregiving styles (*n* = 63)**	
Authoritative (responsive)	23.8% (15)
Indulgent	22.2% (14)
Negligent	14.3% (9)
Authoritarian	39.7% (25)

*Instituto PENSI, 2016*.

The binary logistic regression analysis (Table 2) show that the habit of sharing meals with children was the only factor associated with the maternal caregiving style, and considered a protection factor against nonresponsive care (OR 0.23, 95% CI 0.06–0.88). The other variables were not related to their profile.

**Table 2:** Logistic binary regression according to types of caregiving.

Variable	β	Exp. β	CI 95%
Respect for hunger and satiety signals	0.999	2.71	0.15–49.53
Adequate meal environment	1.29	2.0	0.84–16
Shares meals with child	−1.45	0.23	0.06–0.88
Use of coercive practices	−0.89	0.41	0.12–1.34
Use of distractions at meals	−0.077	1.08	0.18–6.35
Adequate posture at meals	−0.84	0.43	0.12–1.49
Interaction at meals	−0.78	0.46	0.089–2.35
Self-feeding practices	0.066	1.07	0.29–3.85
Adequate use of feeding equipment	−0.75	0.47	0.12–1.90
Prolonged bottle use	−1.50	0.22	0.04–1.19

*Instituto PENSI, 2016*.

*Y, non-responsive caregiving*.

Comparisons between variables according to the four types of caregiving style are described in Table 3. Negligent mothers breastfed exclusively for lower periods of time when compared to authoritarian (diff. 3.6 months, 95% CI 1.68–5.49, *p* = 0.000) and indulgent mothers (dif 3.4 months, 95% CI 0.83–5.94, *p* = 0.007). Mother–child interaction was higher for authoritarian mothers, and lower for negligent ones as well (*p* = 0.001). The remaining variables did not vary according to caregiving styles. After the complementary logistic regression test, mother–child interaction during meals was associated with the authoritarian (OR 0.03 CI 95% 0.004–0.262) and authoritative caregiving styles (OR 0.056 CI 95% 0.006–0.497), both being considered protectors against lack of interaction with children.

**Table 3:** Maternal practices and population characteristics according to caregiving styles.

Variables	Caregiving style [% (*N*) or mean ± SD]				*P*
	Authoritative (responsive)	Indulgent	Negligent	Authoritarian	
Gender					0.26
Females	15.8% (3)	26.3% (5)	26.3% (5)	31.6% (6)	
Male	27.3% (12)	20.5% (9)	9.1% (4)	43.2% (19)	
Age (months)	45.1 ± 24.2	41.6 ± 41.8	43.7 ± 40	45.8 ± 42.7	0.99
Duration of exclusive breastfeeding (months)	2.3 ± 2.3	3.7 ± 2.7	0.29 ± 0.75	3.8 ± 2.9	0.000*
Association with organic disease					0.26
Yes	23.5% (4)	11.8% (2)	23.5% (4)	41.2% (7)	
No	23.3% (10)	25.6% (11)	9.3% (4)	41.9% (18)	
Bottle use					0.05
Inadequate (after 24 months of age)	36% (9)	16% (4)	4% (1)	44% (11)	
Adequate	11.1% (2)	22.2% (4)	27.8% (5)	38.9% (7)	
Self-feeding practices					0.42
Yes	26.5% (9)	26.5% (9)	5.9% (2)	41.2% (14)	
No	27.8% (5)	11.1% (2)	16.7% (3)	44.4% (8)	
Posture at meals					0.51
Adequate	37.5% (6)	18.8% (3)	6.3% (1)	37.5% (6)	
Inadequate	20.5% (9)	22.7% (10)	15.9% (7)	40.9% (18)	
Use of proper utensils					0.47
Adequate	28.6% (12)	16.7% (7)	11.9% (5)	42.9% (18)	
Inadequate	15.8% (3)	31.6% (6)	15.8% (3)	36.8% (7)	
Maternal age (years)	35.4 ± 5.1	37.6 ± 4.9	35.3 ± 6.3	35.5 ± 5.8	0.71
Maternal level of education					0.91
High school	50% (1)	0% (0)	0% (0)	50% (1)	
Superior	22.8% (13)	21.1% (12)	14% (8)	42.1% (24)	
Parity					0.21
Primiparous	25.5% (12)	17% (8)	17% (8)	40.4% (19)	
Multiparous	14.3% (2)	42.9% (6)	7.1% (1)	35.7% (5)	
Coercive practices (use of force)					0.14
Yes	17.9% (7)	17.9% (7)	15.4% (6)	48.7% (19)	
No	34.8% (8)	30.4% (7)	13% (3)	21.7% (5)	
Coercive practices (use of distractions)					0.59
Yes	26.5% (9)	23.5% (8)	8.8% (3)	41.2% (14)	
No	25% (2)	25% (2)	25% (2)	25% (2)	
Duration of meals (min)	33 ± 14.8	75 ± 63.6	20 ± 0.0	43 ± 21.2	0.23
Meal environment					0.21
Adequate	33.3% (8)	20.8% (5)	4.2% (1)	41.7% (10)	
Inadequate	12% (3)	28% (7)	16% (4)	44% (11)	
Presence of adults at meals					0.07
Yes	50% (7)	28.6% (4)	7.1% (1)	14.3% (2)	
No	18.9% (7)	21.6% (8)	16.2% (6)	43.2% (16)	
Shared meals per week	3.6 ± 4.5	2.9 ± 3.9	1.4 ± 2.4	1.5 ± 2.5	0.24
Respect for signals of hunger and satiety (children older than 24 months)					0.50
Yes	50% (1)	0% (0)	0% (0)	50% (1)	
No	26.9% (7)	34.6% (9)	11.5% (3)	26.9% (7)	
Interaction mother–child					0.001
Yes	26.7% (12)	20% (9)	4.4% (2)	48.9% (22)	
No	14.3% (2)	28.6% (4)	42.9% (6)	14.3% (2)	

*Instituto PENSI, 2016*.

*Pearson Chi-squared test*.

*ANOVA test, Tahmane T Post hoc.

## Discussion

Results herein describe the main maternal practices of patients followed at CDA: the responsive profile was characterized by absence of physical coercion to eat, self-feeding practices, use of appropriate feeding equipment and proper meal environments, and presence of interaction during meals. The non-responsive profile was characterized mainly by inadequate posture and meal environment, use of physical coercion and distractions to eat, lack of shared meals, and disrespect to the child’s hunger cues. The non-responsive caregiving profile predominated among mothers, with the authoritarian style as the most prevalent among non-responsive caregiving subtypes. The practice of sharing meals with children was the only factor associated with maternal profile, considered a protective factor against non-responsive caregiving.

Non-responsive caregiving found in the present population is similar to that described by Shloim et al. (20), in a systematic review of 31 articles that discuss parenting styles and responsive practices worldwide. In five studies that address parental styles in this review (using the same classification instrument of the present study), the authors described—among Brazilian and Hispanic immigrants in the US—authoritarian and indulgent parenting styles as the most common. The same profile was described by authors in other studies with non-Latin populations, highlighting a minority of responsive profiles in different ethnicities. In the southeastern region of Brazil, Carvalhaes et al. (21) and Saldan et al. (22) described—in mother–child pairs from low socioeconomic status—a predominance of non-responsive behaviors, such as lack of verbal and low affective contact, low frequency of educational behaviors, presence of threats, distractions, and use of coercion. Such studies were conducted with families without screening for FD, and Carvalhaes et al. (21) pointed out that non-responsive practices were described as even more intense in the presence of refusal to eat. No other studies conducted only with children who presented FD complaints were found, which impairs comparison with present results. Available evidence, therefore, suggests that caregiving profiles tend to be non-responsive in general, regardless of ethnicities and socioeconomic levels.

Silva et al. (2) described the relationship between parental profile and socioeconomic levels as clear and essential: the lower purchasing power, education level, and domestic violence, the higher would be the vulnerability of responsive caregiving. According to the authors, the perception of children’s development, use of nonresponsive practices, and maintenance of adequate caregiving would increase according to education levels. In the present study, however, maternal education levels were not associated with caregiving styles. This relationship may not have been proven due to the population’s uniform profile, mostly with complete superior education and from higher socioeconomic strata (who generally opt for private health services). In addition, both Brazilian studies mentioned above (21, 22) described a non-responsive profile in mothers with low education levels; and a British study with 180 mothers (23) also described an increase in authoritarian practices according to maternal education level. The relationship between caregiving style and the socioeconomic levels remains controversial.

Regardless of these factors, parental psychological characteristics can also be an important influence on responsive caregiving. Elias et al. (24), studying American mothers of lower purchasing power, found that those who reported higher scores on depression symptom scales were at higher chances of non-responsive behaviors (such as pressure to eat and lower perception of cues emitted by the child). According to the psychoanalytic approach, individuals who are in psychological suffering of any kind may become progressively unable to focus on external issues; with an immediate consequence of damage to family dynamics, as well as a disinvestment in the relationship with the child (25). Moreover, current household routines and women’s triple shifts could interfere with the maternal caregiving profiles. These characteristics, however, were not assessed in the present study and cannot be further elucidated.

Comparison between maternal profiles allows inference that all mothers interviewed adopt both proper and inappropriate practices when feeding their children. The difference between profiles regards the frequency of distribution in each group, with a trend toward greater adequacy of behaviors in the group of responsive mothers. Given the mixed set of behaviors described, it could be assumed that all mothers need guidance on their practices, including those considered as responsive caregivers. Regarding interaction with children, responsive caregiving was associated to higher interaction with children (OR 0.056; *p* = 0.01) compared to the authoritarian profile (OR 0.03; *p* = 0.001), suggesting that responsive practices still seem to be the most stimulating. The association between “negligent” profiles and both shorter breastfeeding duration (*p* = 0.000) and interaction with the children (*p* = 0.001) emphasizes that the caregiver’s profile may interfere with early development of the child’s relationship with food, not only when complementary feeding starts. Golen and Ventura (26), for example, described an association between mindless formula feeding and higher volume consumed by infants with low self-regulation capacity; besides mothers who were considered “distracted” presenting less responsiveness to signals emitted by infants (*p* = 0.04). Such data reinforce the need for early identification of parental profiles (since breastfeeding/formulas periods), as subside to encouragement and awareness for early promotion of appropriate behaviors. To that end, there are several validated and available instruments for identification of caregiver’s profile (20, 27–31)—in addition to that used in the present study—that can be combined with multidisciplinary follow-up families.

According to Silva et al. (2), the parental profiles most frequently associated with FD in childhood are “negligent” and/or “authoritarian.” Nevertheless, in the present population, the most frequent non-responsive styles were “authoritarian” and “indulgent,” with “negligent” style being the least prevalent among mothers. There was no difference in feeding practices according to the type of non-responsive care, which might suggest that non-responsive behaviors in general would lead to the same outcome, regardless of their subtype. However, controlled research is essential to confirm these hypotheses. No similar studies were found to allow such comparisons.

As to the possible interventions aiming at correction of non-responsive behaviors, evidence shows that guidance on responsive caregiving promoted increased adequacy of dietary intake, reduced levels of child malnutrition and levels of maternal depression after 10–12 months of follow-up (32, 33); as well as improvement in responsive parental practices and in children’s self-feeding behaviors (34). Overall, interventions to improve quality of mother–infant interactions are based on strengthening parental competencies, including other family members, as well as the mother (35). The present results highlight the need for both psychological and behavioral interventions parallel and simultaneous to traditional multidisciplinary follow-up directed to the family with FD, due to influence of caregiver’s profile and the environment offered for the meals on feeding refusal. These could be both facilitators to the reversal of restrictive eating patterns. Indulgent and negligent caregivers could be encouraged to properly approach children and to get involved with the whole process of feeding their child; while authoritarians and authoritative ones could be taught as to the proper form of interaction with children. Therapy directed to caregiving styles could be a differential factor in the follow-up of children with FD both in outpatient and private services; and would contribute to prevention and management of FD if instituted as a routine guidance primary schools and general multidisciplinary childcare. The Feeding Guide for Children under Two Years of the Brazilian Ministry of Health (36) endorses and addresses these aspects in its 2nd edition, published in 2015.

The interventions on inappropriate behaviors are based on the premises of responsive care, summarized in Chart 1. Both caregiver and child perceive success in their relationship when the meal is offered with proper duration, with reduced child refusal and with low loading of stress, absence of coercive behaviors; and the child exercises independence in manipulating food, and in appetite control, pleasantly interacting with the adult and making eye contact throughout the process in a relaxed and pleasurable posture (2).

**Chart 1:** Guidelines for stimulating responsive behavior.

	Caregiver’s behavior
Non-verbal cues	Recognizes signals emitted by child and responds promptly in the form of support; Smiles, uses words of encouragement, and talks with child about food; Makes eye contact throughout the meal; Feeds the child with disposition, patience, and without haste; Waits for the child to complete chewing and swallowing processes, and to show signs of satiety before offering new portions of food; Provides food that can be manipulated without adult assistance.

Environmental adaptations	Offers meals in proper environments and posture, free from distractions and coercion; Completely involved in the action of feeding the child; The meal happens in the company of family members, preferably eating together.

Food offering	Foods are adequate in consistency, presentation, and nutritional value; offering opportunity to explore flavors and textures.

*Instituto PENSI, 2016*.

Source: Silva et al. (2); Satter (1); Shloim (20)

The present study presents limitations, such as the small size of population, and the absence of psychological and socioeconomic characterization of caregivers. There is need for replication of controlled studies to verify whether FD aggravates maternal behaviors, or if the non-responsive behavior would aggravate the FD itself. The study contributes to the scarce scenario of multidisciplinary follow-up of FD during childhood, creating opportunities for development of educational programs focused on caregivers’ behaviors.

## Conclusion

Non-responsive caregiving profiles were predominantly identified among mothers of children with FD. The habit of sharing meals was considered a protective factor against non-responsive caregiving. The chance of interaction with children increased with responsive practices in comparison to authoritarian style. Results highlight the need for educational interventions focused on caregivers’ behaviors, as well as in-depth case-control studies in families with complaints of FD.

## Ethics Statement

This study was conducted according to the guidelines laid down in the Declaration of Helsinki and all procedures involving human subjects/patients were approved by the Ethical Committee (Instituto PENSI, Brazil), (CAAE 32939314.0.0000.5567; approval granted in 13/08/2014 under document n. 808.394). Written informed consent was obtained from all patients’ parents or legal guardians.

## Author Contributions

RM—main researcher, carried out the study, participated in the design, data analysis, and preparation on manuscript. AB, AT, CR, PM, GM, and LR—assisted in carrying out the study and preparation on manuscript. MF—supervised and assisted all phases of the study (Project PI).

## Conflict of Interest Statement

The PI of the project conferences in events such as Abbott, CPW, EMS, Danone, Nestlé, Nutrociencia, PICME, Sanofi, Wyeth; scientific board member of Danone Institute International, Danone Research, Mondelez. Supports research projects at Abbott, CNPq, Coca-Cola, CPW, Danone Institute International, Danone Research, Fapesp, Fap Unifesp, Nestlé. Authors have no participation in food, nutrition, or pharmaceutical companies, and there is no influence of any company in any of the projects, conferences, or publications conducted. All other authors declare that the research was conducted in the absence of any commercial or financial relationships that could be construed as a potential conflict of interest.
